# Effect of fermentation using different lactic acid bacteria strains on the nutrient components and mineral bioavailability of soybean yogurt alternative

**DOI:** 10.3389/fnut.2023.1198456

**Published:** 2023-06-23

**Authors:** Jing Gan, Xiao Kong, Kuaitian Wang, Yuhang Chen, Mengdi Du, Bo Xu, Jingru Xu, Zhenhua Wang, Yongqiang Cheng, Tianying Yu

**Affiliations:** ^1^Center for Mitochondria and Healthy Aging, College of Life Science, Yantai University, Yantai, Shandong, China; ^2^Beijing Key Laboratory of Functional Food from Plant Resources, College of Food Science and Nutrition Engineering, China Agricultural University, Beijing, China

**Keywords:** mineral, speciation analysis, absorptivity, zebrafish model, fermentation, yogurt alternative

## Abstract

**Introduction:**

Analysis of the composition of yogurt alternatives (YAs) during fermentation provides critical information for evaluating its quality and nutritional attributes.

**Method:**

We investigated the effects of homotypic (HO) and heterotypic (HE) lactic acid bacteria on the nutritional and mineral bioavailabilities of soybean YA (SYA) during fermentation.

**Result:**

The acidic amino acid (Glu, Asp) and organic acid contents in HO-fermented YA were increased from 2.93, 1.71, and 7.43 mg/100 g to 3.23, 1.82, and 73.47 mg/100 g, respectively. Moreover, both HO and HE lactic acid bacteria fermentation enhanced mineral absorptivity. They altered the molecular speciation of minerals from a large molecular type (2,866 Da) to a small molecular type (1,500 Da), which was manifested in a time-dependent manner. Furthermore, YA substantially increased the bone mass in a zebrafish osteoporosis model, further highlighting the potential of lactic acid bacterial fermentation for mineral bioavailability.

**Discussion:**

This study provides a foundation for understanding the effects of fermentation conditions on the composition and bioavailability of minerals in YA and can assist in its production.

## 1. Introduction

Soybean milk, obtained by crushing and pressing the water-soaked soybean, is a popular soybean-derived food (1). Daily intake of soybean milk has a favorable effect on cardiovascular, glycemic status, and blood pressure (2, 3). Soybean milk, is considered an alternative to cow’s milk and is widely consumed in populations suffering from lactose intolerance and milk proteins allergies (4).

Soybean milk is a primary source of mineral elements that play essential roles in sustaining life and supporting fundamental body functions (5–10). For example, calcium regulates the activity of intracellular enzymes and participates in neuronal conduction via ion channels. Magnesium is required for enzymatic activity and is critical for DNA and RNA synthesis, reproduction, and protein synthesis. Ferric iron is a trace mineral involved in oxygen transport and cellular respiration. Calcium, magnesium, and phosphorus are involved in bone tissue formation. Generally, efficient absorption is essential for performing functional activities in the body (11), but the speciation of minerals limits their absorption in the gastrointestinal environment (12). A previous study demonstrated that the absorptivity differed among the carbonate, pyruvate, and citrate/malate formulations of calcium. Among these, the carbonate and pyruvate formulations were more effective in calcium supplementation (13). Several studies have shown that organic trace elements improve membrane permeability at a higher rate than inorganic forms due to their higher lipophilicity, making them better absorbed and utilized (14). However, Liu et al. reported that bioactive gelatin peptide-chelated calcium had a higher utilization rate and absorptivity than inorganic and organic calcium (15). Nonetheless, studies have also shown that major anti-absorptive factors such as phytate, oxalate, uric acid, and dietary fiber in soy milk decrease the absorption of the minerals (16). Furthermore, (17–18) showed that minerals in food, such as Na and K, Ca and Mg, Mn and Fe, and Fe and Cu, might inhibit their absorption and utilization rates. This mutual inhibition effect between minerals has been speculated to be related to their shared absorption through cell membranes. Therefore, understanding the forms of mineral elements in foods can improve their bioavailability of mineral elements.

In recent years, yogurt alternatives fermented by lactic acid bacteria have effectively enhanced the number of bioactive ingredients and flavors in soy milk and have attracted increasing attention. In our preliminary experiments, we found that the absorption rate of calcium significantly increased in yogurt alternatives, which could be related to changes in the mineral element morphology during fermentation. However, it is not clear whether the absorptivity of other mineral elements besides calcium increases during fermentation. Furthermore, the relationship between bacterial strains, mineral morphology, and absorption remains unclear. Health problems are highly related to diet and nutritional habits (19). Therefore, we speculated that systematic research to elucidate the effects of speciation on mineral absorption in yogurt alternatives produced using different strains could provide better insights.

To test this speculation, the present study aimed to investigate the absorptivity and speciation of minerals in YAs produced using different strains of lactic acid bacteria. Here, we prepared two YA samples by fermenting soybean milk with two different lactic acid bacteria and analyzed their absorptivity *in vitro* and *in vivo*. The molecular weight distribution and the speciation of minerals in the YA samples were characterized by protein structural changes, amino acid production of the strains, and the release of organic acids during fermentation.

## 2. Materials and methods

### 2.1. Starter cultures and soybean

Pure freeze-dried thermophilic yogurt cultures for the direct-to-vat set form, *Lactiplantibacillus plantarum* CICC 21022 and *Enterococcus faecalis* ATCC19433were screened in our laboratory. Briefly, 5 g of pickled Chinese cabbage was washed five times with sterile water. Serial decimal dilutions were obtained, plated on MRS agar supplemented with 2% calcium carbonate, and then incubated under aerobic and anaerobic conditions at 37°C for 48 h. Gram-positive, catalase-negative isolates were considered presumptive lactic acid bacteria. Subsequently, the 16S rDNA segments were amplified and sequenced to identify the structure of lactic acid bacteria. Soybean was purchased from the Chinese Academy of Agricultural Sciences. Fetal bovine serum (FBS), Dulbecco’s Modified Eagle’s medium (DMEM), and other reagents used for cell culture were purchased from Corning (NY, USA). All other reagents were either analytical grade or pure (Solarbio Life Sciences, Beijing, China).

### 2.2. Preparation of soybean milk and soy yougurt

YA was successfully prepared using a previously reported method (20) with slight modifications. Briefly, soybeans were soaked in water at a ratio of 1:3 (soybean: water) at room temperature for 12 h, followed by hot grinding (a volume 12 times higher than that for soybeans) and remove soybean residue through a 100 mesh sieve. After adding 2% (w/v) sugar in 100 ml soybean milk, the soybean milk was sterilized using high-pressure sterilization at 220 bar for 15 min. For the fermentation process, the *Lactiplantibacillus plantarum* CICC 21022 and *Enterococcus faecalis* ATCC19433 were cultured with 7.512 × 10^7^ CFU/ml, following incubate at 37°C at facultative anaerobic conditions for 12 h, the SYA was collected and stored at −20°C for subsequent use.

### 2.3. Microbiological analysis

The amount [colony-forming units per ml (CFU/ml)] of *L. plantarum* and *E. faecalis* populations was determined by plating the aliquots of the serial dilution on MRS agar, and incubating at 37°C for 48 h at facultative anaerobic conditions. The aliquots of the serial dilution was plated on MRS agar, and incubated at 37°C for 48 h under the conditions described above. The colony-forming units per ml (CFU/ml) of *L. plantarum* and *E. faecalis* populations was determined following the official microbiological methods.

### 2.4. Analysis of protein composition during fermentation

#### 2.4.1. Detection of protein content and amino acid profile

The total soluble protein content was determined using bicinchoninic acid (BCA) assay. All samples were combined with 6 M HCl and left to hydrolyze for 4 h at 110°C. An automatic amino acid analyzer (835–50, Hitachi LA8080) was used to determine the amino acid composition. Briefly, 1 g of soybean milk or YAs fermented by different lactic acid bacteria was dissolved in 25 ml ultrapure water. Subsequently, 0.5 ml potassium ferrocyanide (106 g/L) and zinc sulfate (300 g/L) each were added to the solution and allowed to flow statically for 1 h. The separation was performed using a CAPCELL PAK C18 column (150 mm × 4.6 mm × 3 μm) and sodium dihydrogen phosphate solution (20 mmol/L) mobile phase under the following conditions: flow rate, 0.9 ml/min; detection wavelength, 210 nm; column temperature, 30°C; sample injection volume, 10 μl. The sample values were determined based on a calibration curve developed using five standards (Asp, Glu, Leu, His, and Arg).

#### 2.4.2. Sodium dodecyl sulfate-polyacrylamide gel electrophoresis

SDS-PAGE was performed following a previously reported method (21) to analyze the primary structure of the protein. PAGE was performed using 12% concentrated and 4% separated glues at a loading amount of 20 μl.

### 2.5. Determination of sugar and organic acid content during fermentation

#### 2.5.1. Determination of sugar content

Changes in free oligosaccharide content were detected using ion chromatography. Briefly, 0.2 g soybean yogurt was dissolved in 10 ml water and diluted to 25 ml by water. The chromatographic separation was performed using a DIONEX ICS-3000 USA system fitted with a Carbo PacTMPA20 3*150 mm analytical column (Diane Corporation of the United States), a pulse ampere detector, and gold electrode under the following conditions: Eluent solution, 250 mM NaOH; flow rate, 0.5 ml/min; injection volume, 10 μl. The separation was carried out under gradient elution conditions using pure water (solvent A) and 250 mM NaOH (Solvent B) as follows: 0–20 min, 94% A, 6% B; 20–20.1 min, 20% A, 80% B; 20.1–30 min, 20% A, 80% B, 30.1–40 min, 94% A, 6% B.

#### 2.5.2. Determination of organic acid content

The organic acid content was determined using HPLC. The chromatography was performed using a CAPCELL PAK C18 (150 mm × 4.6 mm × 3 μm) LC column and sodium dihydrogen phosphate solution (20 mmol/L) at a flow rate of 0.9 ml/min as the mobile phase. A sample volume of 10 μl was injected, and the signals were detected using a UV detector at 215 and 210 nm. Oxalic, tartaric, lactic, acetic, citric, succinic, malic, and ascorbic acids were used as standard solutions to develop the calibration curve. Briefly, One gram sample was dissolved in 15 ml buffer containing 0.5 ml potassium ferrocyanide (106 g/L) and 0.5 ml zinc sulfate (300 g/L) and allowed to stand for 1 h at room temperture. Afterward, the sample was centrifuged at 7104 × *g* and analyzed, individually.

### 2.6. Determination of the content of minerals, their speciation, and absorptive rate during fermentation

#### 2.6.1. Determination of mineral (Ca, Zn, Mg, Fe, P) content of soy milk during fermentation

Soybean milk or YA samples (0.20 g) were weighed (accurate to 0.0001 g), placed in a Teflon (Polytetrafluoroethylene; PTFE) tank, combined with concentrated HNO_3_ (3:1), and left to stand for 4 h. Then, the PTFE tank was placed into a stainless steel coat and heated for 4 h at 165°C. The Ca, Zn, Mg, Fe, and P contents of the samples were determined using inductively coupled atomic emission spectrometry (ICP-AES; Prodigy xp, GmbH). The experimental conditions, including the auxiliary and gas flow rates, cooling gas flow, and generator power, were 0.8 L/min, 0.8 L/min, 12 L/min, and 1.4 kW, respectively.

#### 2.6.2. Mineral speciation analysis of soybean milk during fermentation

HPLC, in combination with inductively coupled plasma-mass spectrometry (ICP-MS), was used to detect mineral and trace element species in YA samples during fermentation. Samples were separated using an XBridge Protein BEH SEC 200 Å column (7.8 mm × 300 mm × 3.5 μm). Briefly, the samples (50 mg) were dissolved in 0.03 mol/L Tris–HCl buffer (pH 7.4) and filtered using a 0.45 m filter membrane. The sample (20 μl) was injected and separated using – mobile phase at a flow rate of 0.5 ml/min; the detection wavelength was 214 nm (UV detector). The analysis was performed with external calibration by developing the calibration curve derived from five standards: BSA (66.4 kDa), trypsin inhibitor (20.1 kDa), insulin (5.8 kDa), VB12 (1.35 kDa), and L-tyrosine (0.187 kDa). The molecular weight of the sample peaks was determined based on that of the standards.

#### 2.6.3. Cell culture, transport assays, and calculation of apparent permeability

The human colon carcinoma cell line, Caco-2, was used as an intestinal barrier model. Transport assays were performed in Transwell systems using the method described by Wang et al. (22). Three transport sides were tested: apical, intermediate, and basal. The Transwell layer was rinsed twice with standard solutions of Hank’s balanced salt solution (HBSS) without magnesium and calcium. Caco-2 cells were laid as the intermediate layer and washed twice using HBSS. Subsequently, 1.5 and 0.5 ml of HBSS were added on the basolateral and apical sides, respectively, and the cells were preincubated for 30 min at 37°C. The absorption assays were performed by adding 0.5 ml sample fractions (0.1 mg/ml) and replenishing the HBSS on the apical side. Afterward, the cells were cultured at 37°C for 2 h. The apical, intermediate, and basal fluids were collected according to the method described in Table 1. The mineral content was determined using ICP-AES (ICAP-6300, Thermo Electron Corporation, USA). The calcium absorption was calculated using the following formula:


(1) $$\text{Absorption of mineral}\;\left(\%\right)=\left[\begin{array}{l} \left(C_{\text{Basal}}\times V_{\text{Basal}}\right)+\\ \left(C_{\text{Intermediate}}\times V_{\text{Intermediate}}\right)/\\ \left(C_{\text{Apical}}\times V_{\text{Apical}}\right)+\\ \left(C_{\text{Basal}}\times V_{\text{Basal}}\right)+\\ \left(C_{\text{Intermediate}}\times V_{\text{Intermediate}}\right) \end{array}\right]\times 100\%$$

**Table 1:** Changes of free oligosaccharide in soybean milk fermented by *Lactiplantibacillus plantarum* CICC21022 and *Enterococcus faecalis* ATCC19433.

Free oligosaccharide	Composition (%)		
	Soybean milk	Yogurt alternative_(LP)_	Yogurt alternative_(EF)_
Glucose	0.02	0.03	0.03
Sucrose	47.29	26.47	28.05
Fructose	0.03	0.04	0.04
Raffinose	0.09	0.14	0.21
Stachyose	23.00	19.80	15.53

C_Basal_ indicates the calcium concentration at different time points on the basal interior side; V_Basal_ denotes the volume corresponding to the time points on the inner basal side; C_Intermediate_ and C_Apical_ represent the mineral concentrations on the middle and apical sides, respectively; V_Intermediate_ and V_Apical_ denote the corresponding volumes at the middle and apical sides, respectively.

#### 2.6.4. Absorptivity clustering analysis of minerals in YA

We obtained the data on mineral absorptivity, Bacterial strain, and mineral type were collected and subjected to orthogonal transformation analysis using SPSS 16 software. A group of variables speculated to be correlated was converted into linearly unrelated variables to reduce the dimension while maintaining the characteristics to which the variance contributes the most. Then, we performed principal component analysis (PCA) using the ‘vegan’ package based on the Bray–Curtis distance on the R platform to analyze the correlations and trends of the data. The outputs were obtained in a grid, and the color-synthesis results of the first and second principal components were previewed.

### 2.7. Evaluation of fermentation broth against glucocorticoid-induced osteoporosis using Zebrafish larva

#### 2.7.1. Zebrafish maintenance and fermentation broth treatment

All the procedures were performed in accordance with the National Institutes of Health Guidelines for the Care and Use of Laboratory Animals. Adult zebrafish AB strains were kept at 28.5°C under a normal light/dark cycle of 14 h/10 h at 28°C for natural maturation. Zebrafish embryos were cultured in 6-well plates. The collected embryos were placed in fish water containing 5.0 mM NaCl, 0.17 mM KCl, 0.33 mM CaCl_2_, and 0.33 mM MgSO_4_ and treated with 0.1% DMSO (vehicle), 25 μM prednisolone (model), or co-incubated 3.3–30 μM CBP, or 0.308 μM alendronate (positive control) after 3 pdf till 7 dpf. On pdf 7, larvae were collected for skeletal staining and behavioral analysis.

#### 2.7.2. Vital skeletal staining and image acquisition

The fish at 7 dpf were vitally stained with 0.2% calcein solution (pH adjusted to 7.0–7.5) for 5 min in the dark. Next, fish were rinsed thrice for 5 min with the system water, anesthetized with 0.016% MS-222, and fixed in 3% methylcellulose. At the end of the treatment, the regenerated bone areas on the larvae were visualized using a fluorescence microscope (Leica DMi8, Wetzlar, Germany), and images were captured using digital cameras (10X objective, and Retiga 2000R). The relative fluorescence intensity (RFI) of skull bone mass per zebrafish was determined using ImageJ software (n = 6).

### 2.8. Statistical analyses

All tests were performed at least in triplicates in six independent experiments. The results were subjected to statistical analysis using one-way ANOVA with Duncan’s posthoc test for comparison with the vehicle. All figures were drawn using GraphPad Prism 5.0 (GraphPad Software, San Diego, CA, USA) and Excel (Microsoft, San Francisco, CA, USA). Differences at a *p*-value of <0.05 were considered statistically significant.

## 3. Results

### 3.1. Acidity of YA, free oligosaccharide, and availability of lactic acid bacteria culture during fermentation

**Figure 1:**
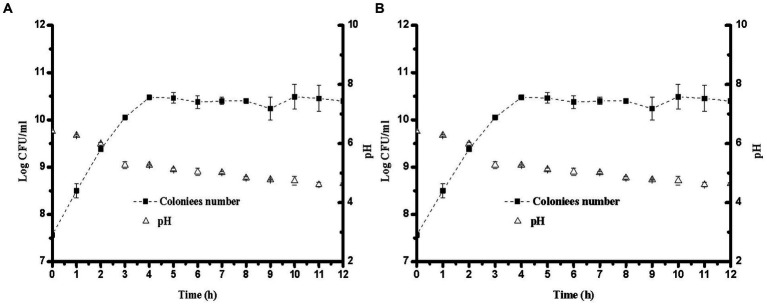
Growth curve and pH for different lactobacillus in soybean milk at 37°C over 12 h. **(A)** *Lactiplantibacillus plantarum* CICC 21022; **(B)** *Enterococcus faecalis* ATCC19433.

**Figure 2:**
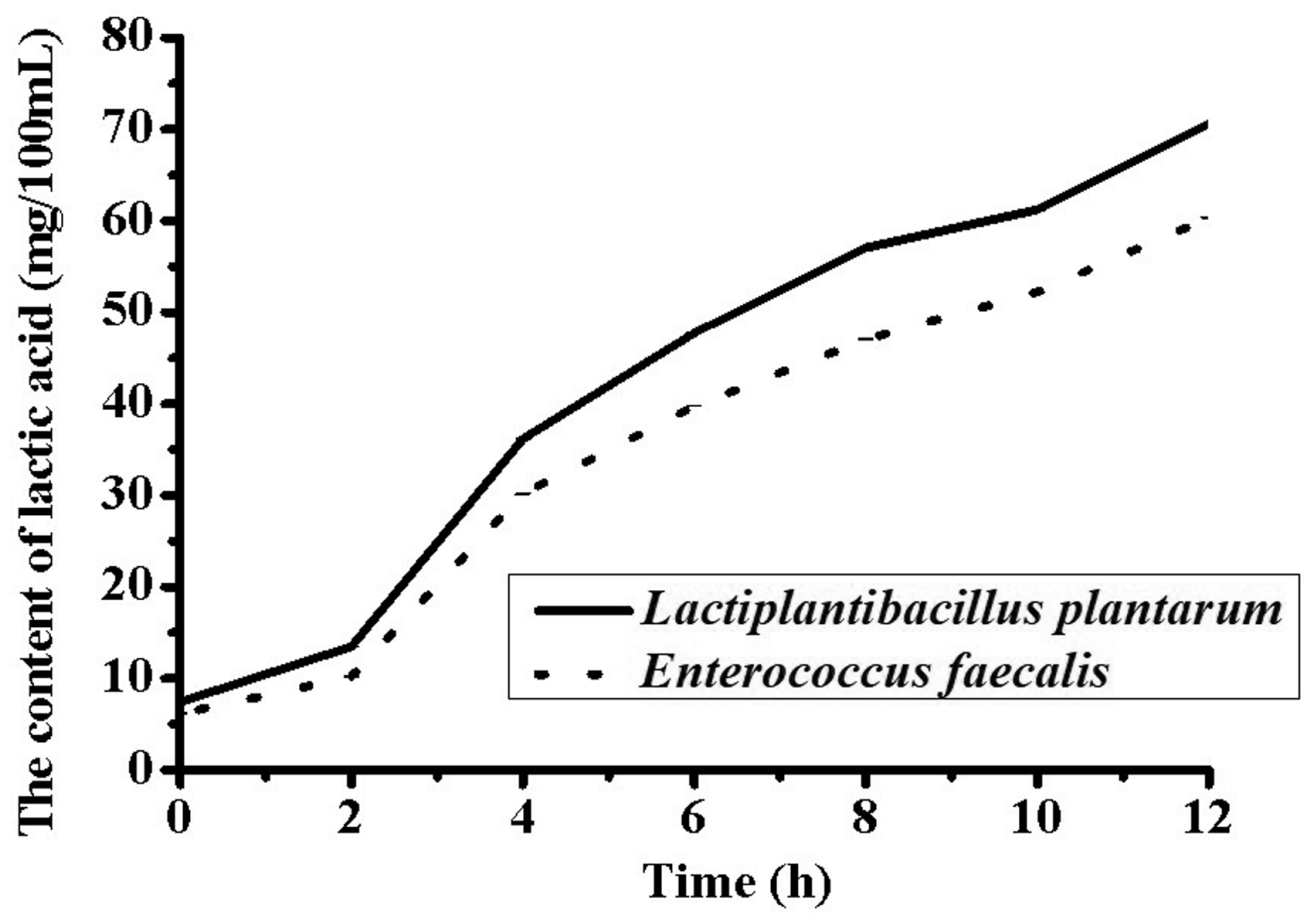
Changes of Organic acid in soy yogurt fermented by *Lactiplantibacillus plantarum* CICC 21022 and *Enterococcus faecalis* ATCC19433.

Changes in pH during fermentation with the two different strains are shown in Figure 1. The largest decrease in pH occurred at 3 h, and the pH range of 4.6–4.7 was obtained after 8 h of fermentation. This phenomenon might be due to the growth of *L. plantarum* and *E. faecalis*, which produce more lactic acid from sugar (23). Compared to *E. faecalis*, *L. plantarum* showed faster acid-producing characteristics. To improve the growth characteristics of lactic acid bacteria during the initial fermentation stage, sucrose was added to soybean milk. The total sugar content in the YA at 0 h was higher than that in soybean milk. However, the proportion of threose decreased, possibly due to the decomposition of raffinose and stachyose. As the pH decreased, *L. plantarum* and *E. faecalis* grew faster, and *L. plantarum* showed higher acid tolerance than *E. faecalis*. The lactic acid contents of soybean milk samples containing different strains after 12 h of fermentation are shown in Figure 2. Increasing the fermentation time significantly affected the formation of acetic acid in soybean milk. Furthermore, consistent with the changes in pH, the lactic acid content of *L. plantarum* was higher than that of *E. faecalis*.

### 3.2. Effect of fermentation on YA components

#### 3.2.1. Effect of fermentation on protein composition

**Figure 3:**
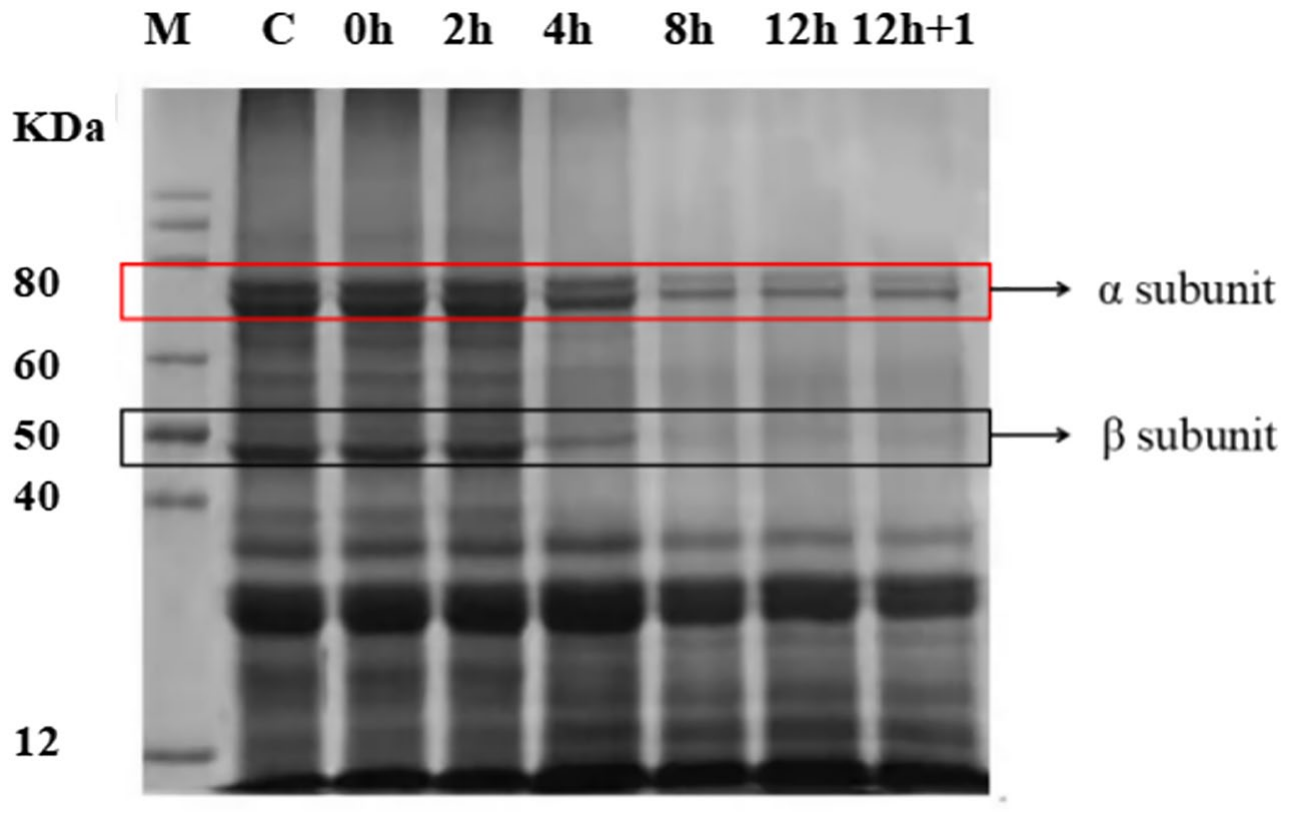
Sodium dodecyl sulfate-polyacrylamide gel electrophoresis (SDS-PAGE) analysis of the changes of soybean milk protein during fermentation by *Lactiplantibacillus plantarum* CICC 21022.

The analysis of the changes in soluble protein in YA fermented by *L. plantarum* revealed a sharp decrease in the amount of soluble protein from 20.3016 to 1.71 μg/ml at first 4 h, which reached 1.33 μg/ml at rock bottom. Furthermore, SDS-PAGE analysis to characterize the polypeptide composition of the globulin fractions identified 7S as the main fraction with α (67 kDa), α′(71 kDa), and β (45–50 kDa) subunits (Figure 3). In addition, 11S, with AS (32 kDa) and BS (28 kDa) subunits, was also identified as a minor fraction. The SDS-PAGE patterns did not change until 4 h of fermentation, indicating that the subunits remained intact within this time point. However, at 8 h, the band intensities of 7S and 11S globulins decreased slightly. After 12 h, the 7S globulin bands largely disappeared, and the basic subunit of 11S globulin was fainter than the initial one. These changes in protein content and structure observed in *L. plantarum*-fermented SYA were similar to those in *E. faecalis*-fermented SYA (Supplementary Figure S1).

#### 3.2.2. Effect of fermentation on amino acid composition

The amino acid order and content determine the shape and functional activity of the protein (24). Changes in amino acid composition were analyzed to explore the relationship between the mineral components and bioactivity during fermentation. As shown in Table 2, the content of individual amino acids increased after fermentation, among which Glu was the most abundant, followed by Asp. The acidic amino acid contents of Glu and Asp in soybean milk and YA fermented with *L. plantarum* increased by 2.93 to 3.23 and 1.71 to 1.82, respectively. These changes were also observed in soybean milk and SYA fermented with *E. faecalis*.

**Table 2:** The composition of amino acid in soybean milk and Yogurt alternatinve fermented by *Lactiplantibacillus plantarum* CICC21022 and *Enterococcus faecalis* ATCC19433 for 12 h.

Amino acid	Composition (%)		
	Soybean milk	Yogurt alternative_(LP)_	Yogurt alternative_(EF)_
Asp	17.19	18.19	18.92
Thr	5.06	5.8	5.58
Ser	6.63	7.43	7.29
Glu	29.31	32.34	32.50
Gly	5.61	6.36	6.21
Ala	5.65	6.41	6.27
Cys	0.72	0.99	0.71
Val	6.43	7.23	7.16
Met	1.30	1.62	1.43
Ile	6.27	7.08	6.99
Leu	10.22	11.53	11.40
Tyr	3.66	4.59	3.34
Phe	7.10	7.93	7.88
Lys	8.54	9.53	9.49
His	3.56	3.98	3.93
Ar	12.72	11.13	11.86
Pro	6.96	7.84	8.05

A. Yogurt Alternative_(LP)_: the yogurt alternative feremened by *Lactobacillus plantarum* for 12 h; B. Y ogurt Alternative_(EF)_: the yogurt alternative feremened by *Enterococcus faecalis* for 12 h.

### 3.3. Contribution of fermentation to mineral absorptivity

#### 3.3.1. Analysis of total mineral absorption ratio

**Figure 4:**
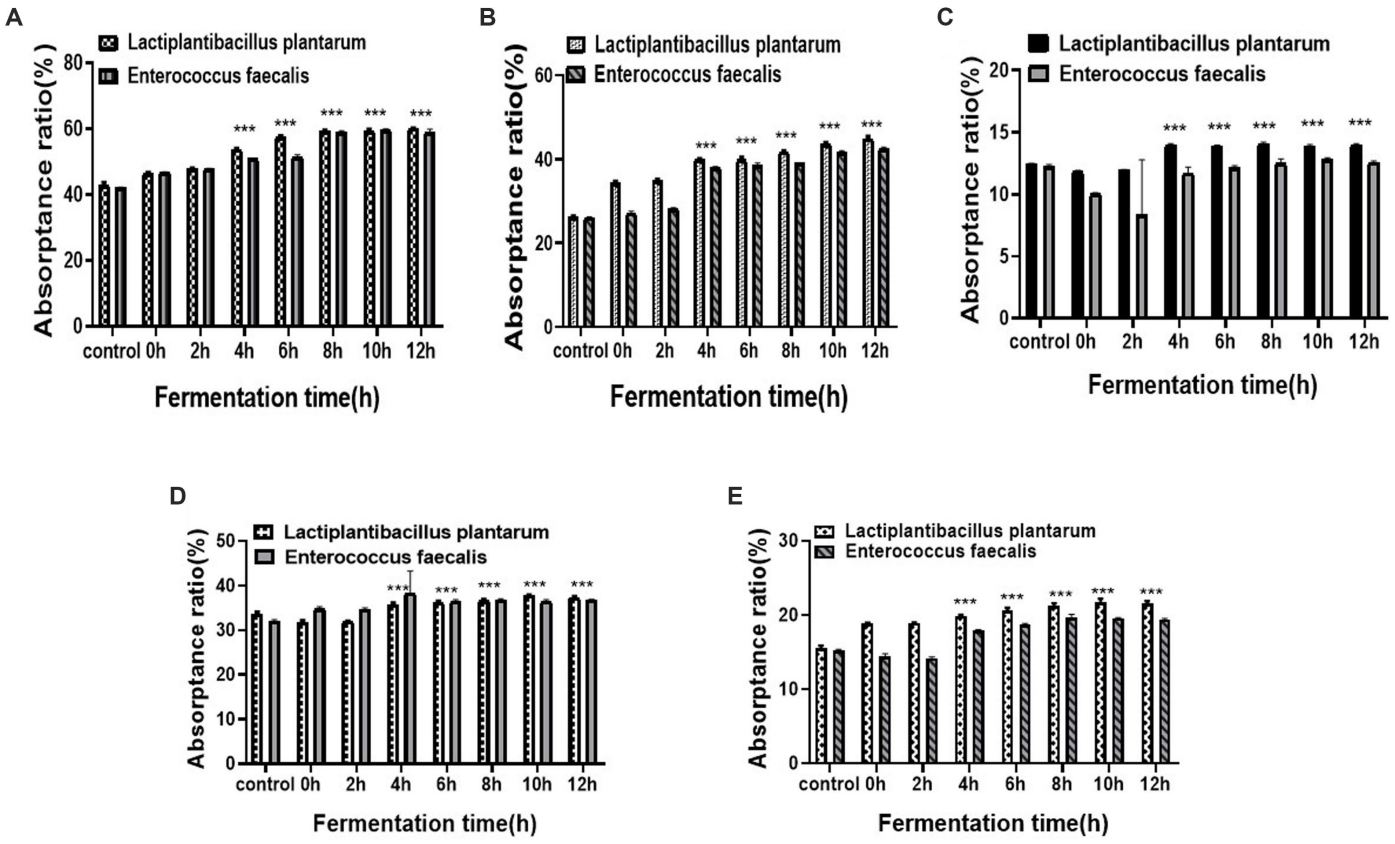
Total mineral absorptance ratio over different fermentation times **(A)** Calcium; **(B)** Ferrum; **(C)** Magnesium; **(D)** Zinc; **(E)** Phosphorus. Data are presented as means ± SEDs and analyzed by one-way ANOVA followed by Tukey’s multiple comparison test **p < 0.01, and ***p < 0.001 vs. control.

Changes in the total mineral absorption ratio during fermentation are shown in Figure 4. For *L. plantarum* and *E. faecalis* inoculations, the detection of total minerals was most evident in a time-dependent manner in all treatment groups. Both *L. plantarum* and *E. faecalis* significantly increased the absorption rate from 4 to 8 h and reached a maximum at 12 h, with a calcium absorption rate of 60%. At 4 h, the calcium absorption rate of *L. plantarum*-fermented YA was higher (25%) than that of *E. faecalis*-fermented YA (21%). A similar trend of increased absorption in *L. plantarum*-fermented YA compared to that in *E. faecalis*-fermented YA was observed in absorption of ferrum, zinc, magnesium, and phosphorus, which declined up to 2 h, and increased thereafter.

#### 3.3.2. Analysis of contribution to mineral absorption by different bacteria

**Figure 5:**
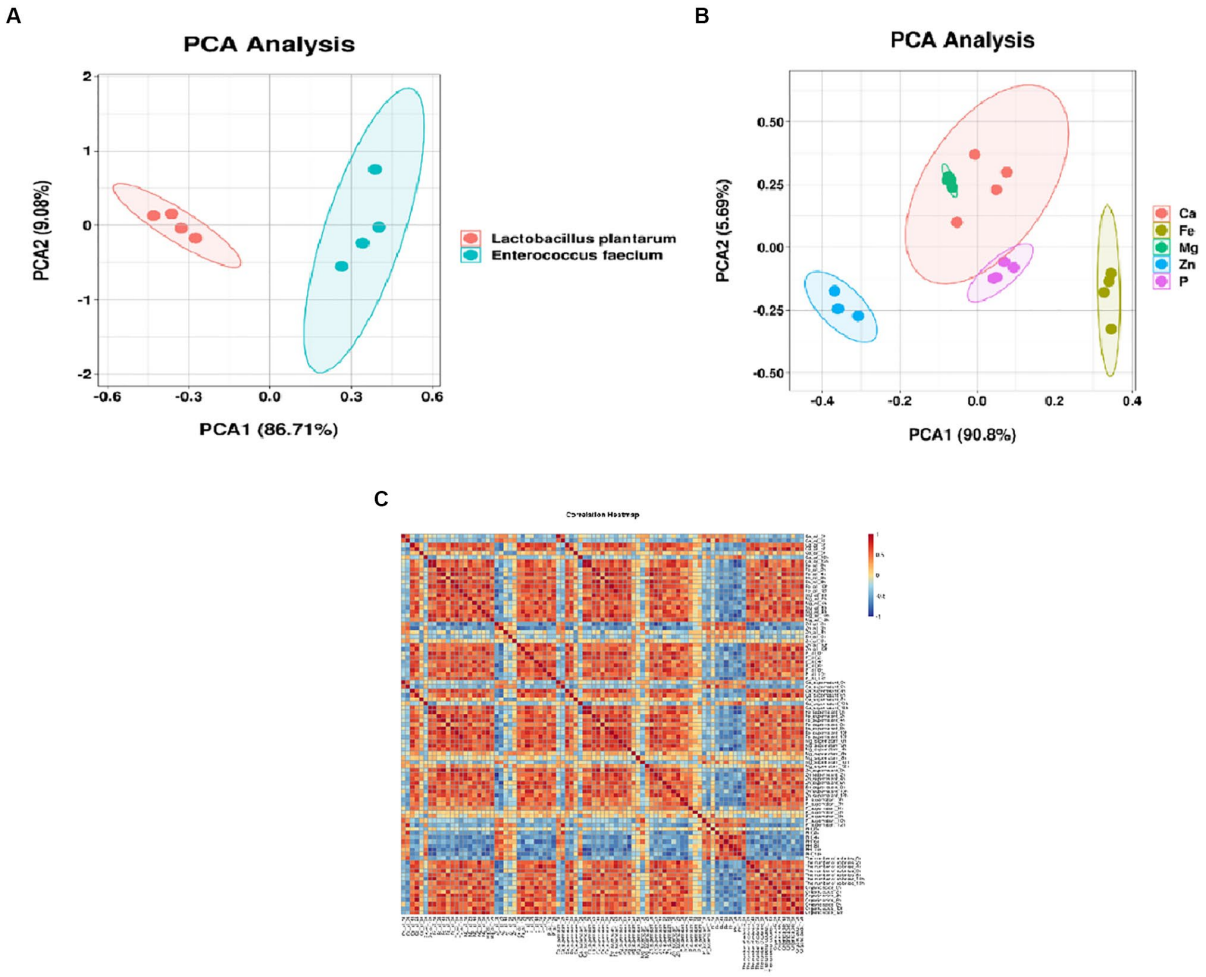
Absorptivity clustering analysis of minerals in soy yogurt **(A)** different strains; **(B)** different minerals; **(C)** correction analysis of strains, kinds and absorptivity of soy yogurt).

Different stains Absorptivity clustering analysis of minerals in soy yogurt fermented by different strains is shown in Figure 5A. Figure 5B shows the distribution of the samples as a function of the two principal components extracted by PCA analysis, which accounted for 96.49%, presenting the preliminary information about the sample. Additionally, there were a few differences between calcium absorption and iron absorption according to the apparent overlap. The other elements were far apart, and the parallelism of each element was close, demonstrating a significant difference and comprehensive repetition. In the correlation analysis (Figure 5C), different degrees of correlation were observed among various attributes. The pH values were negatively correlated with other parameters. At the same time, the absorption rate of minerals was positively correlated with fermentation time, number of colonies, and organic acid content. These results agree with the absorption ratio data. In summary, the absorption profiles of YAs can be comprehensively evaluated by measuring these components.

#### 3.3.3. Analysis of mineral speciation

**Figure 6:**
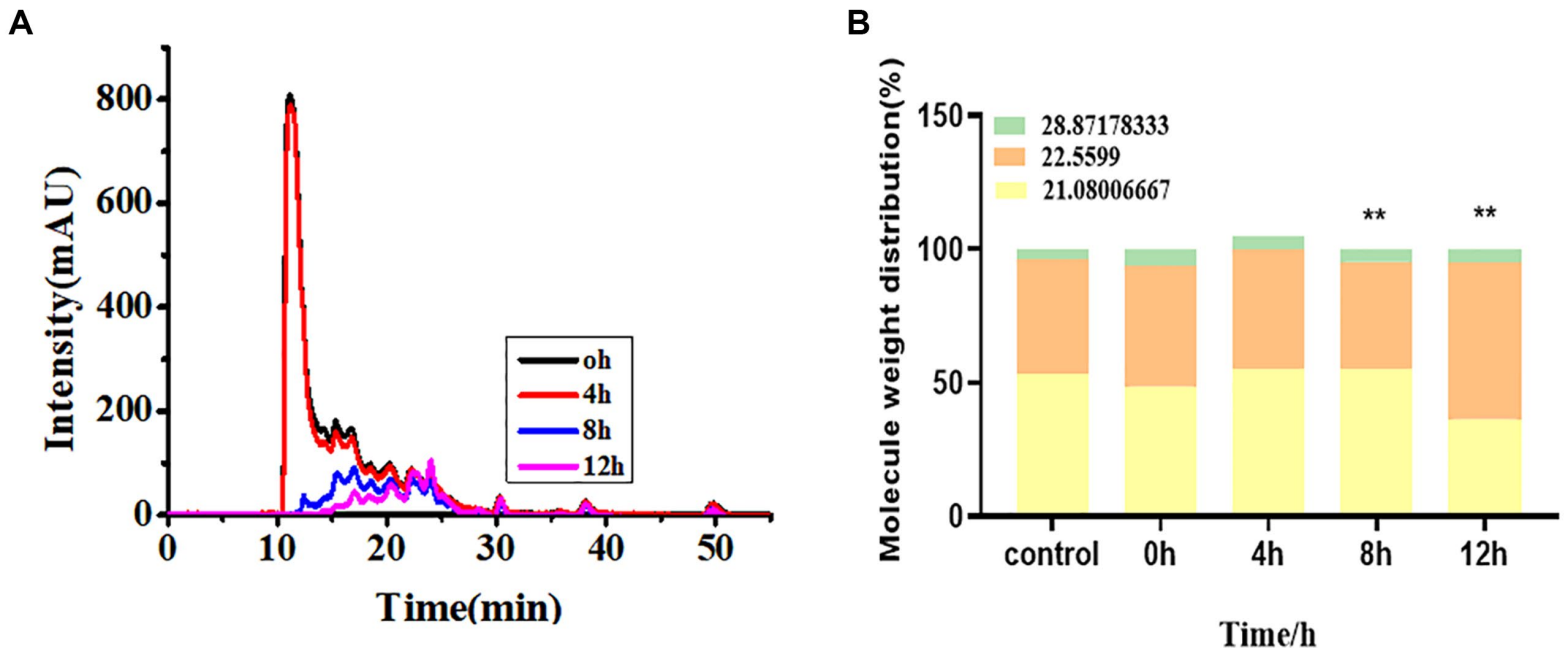
**(A)** Chemical form changes, **(B)** molecular weight distribution of hydrolysate over different fermentation times.

The molecular weight distribution and morphology distribution of minerals in soybean milk during fermentation by *L. plantarum* are shown in Figure 6. Most molecules were isolated at 11.56 min with a total molecule weight of 170 kDa. The molecular weight of individual molecules ranged from 1 to 63 kDa. The inoculation of fermentation strains into soybean milk increased the abundance of low-molecular-weight minerals with a significant increase at 4 h; this was stabilized at 10 h because of protein flocculation and precipitation. Additionally, the lower molecular weight (1,500 Da) signal increased after 6 h, which suggests that more acidic amino acids provide mineral binding sites, thus leading to high bioavailability.

Next, to understand the effect of the form of the mineral on bioavailability, we evaluated the possible mineral forms using HPLC-ICP-MS. As shown in Figure 6B, the elution of three calcium peaks was observed with retention times of 21.08, 22.56, and 28.87 min, corresponding to molecular weights of 2,866, 1,532, and 102.8 Da, respectively. A few peaks were observed at 0 h and 2 h 12 min, which disappeared after 4 h. Nevertheless, the signal of the lower molecule weight (1,500 Da) increased after 6 h, which suggests more hydrolyzed acidic amino acids provide a calcium-binding site, thus leading to high bioavailability. Morphological changes in iron, magnesium, zinc, and phosphorus during fermentation are shown in Supplementary Figure S2. Soybean milk fermented with *E. faecalis* exhibited a similar trend.

### 3.4. Effect of YA on bioavailability *in vivo*

**Figure 7:**
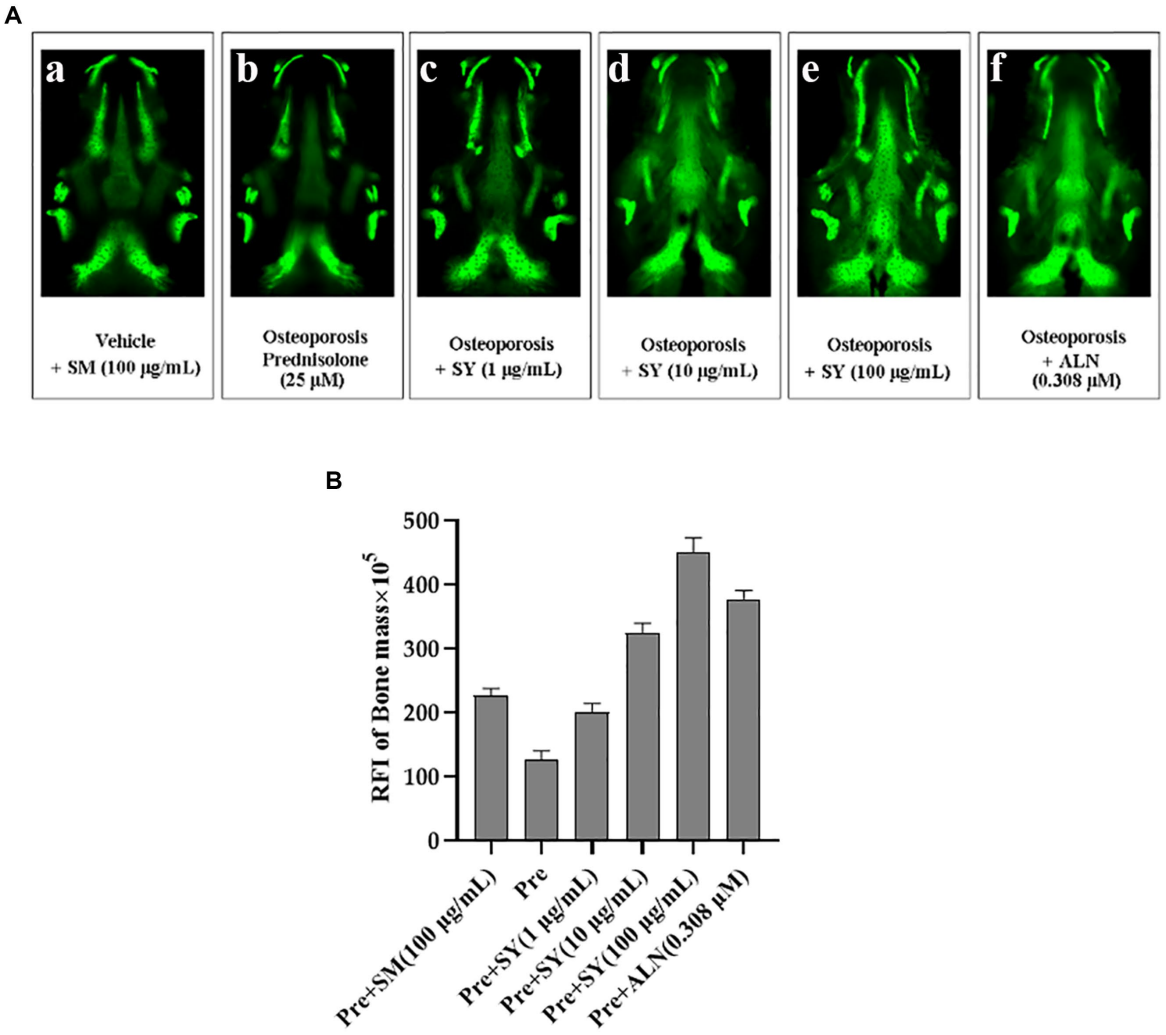
Effects of soy yogurt on anti-osteoporosis in the zebrafish model of GIOP. **(A)** Quantification of the relative fluorescence intensity (RFI) of the zebrafish skull at 7 dpf. **(B)** Representative fluorescence images of the zebrafish skull with different samples at 7 dpf (a) 0.1% DMSO (Vehicle), (b) 100 μg/ml prednisolone (Osteoporosis), or co-administered with soy yogurt (1 μg/ml, 10 μg/ml, 100 μg/ml; c–e), or 0.308 μM alendronate (positive control; f) for 96 h. Data are expressed as mean ± SEM. ^##^*p* < 0.01, ^###^*p* < 0.001 vs. Vehicle, *p < 0.05, **p < 0.01, and ***p < 0.001 vs. Pre, ns, not significant (Pre, prednisolone; ALN, alendronate).

Zebrafish have become an attractive animal model for studying mineral bioavailability because of their small size, short developmental cycle, and transparent embryos. Calcium is essential for the prevention of osteoporosis, and adequate intakes of zinc, magnesium, magnesium, and phosphorus are also presumed to play important roles in osteoporosis prevention (25). To investigate the preventive effects of yogurt on mineral bioavailability, we recapitulated prednisolone(PSL)-induced osteoporosis-like phenotypes. In addition, calcein, as a green fluorescent chromophore, specifically binds to calcium used for vital staining (26). Compared to the control group, prednisolone exposure caused a significant decrease in the stained mineralized larval tissue. In a well-established model, we observed a reduction in fluorescence intensity in Alendronate (ALN; 0.308 μM) and fermentation broth (*L. plantarum*) treatments, which reversed (Glucocorticoid Induced Osteoporosis)GIOP-induced bone loss, as shown in Figure 7. Quantitative analysis using the ImageJ software revealed that the RFI of zebrafish in the model group was lower than that in the control group. Moreover, in the fermentation treatment group, a significant increase was observed in the RFI of bone mass compared with that in the prednisolone treatment group, reaching 28% SYA treatment group at 100 μg/ml. These results indicate that the enhancement of the absorption rate of the YA can prevent bone loss symptoms in the skulls of zebrafish.

## 4. Discussion

Yogurt, a fermented milk product obtained by lactic acid bacteria-mediated acidification of milk, is consumed widely in developing and developed countries due to its functional activities (27). However, an increasing number of people expect to replace yogurt with plant-fermented beverages because of problems such as allergenicity and the desire for vegetarian alternatives (28). SYA, obtained by fermenting soybean milk using lactic acid bacteria, has gained increased interest because of its rich protein content, multiple mineral elements, and low price. Moreover, several studies have demonstrated its potential as an excellent alternative to cow’s milk (29). However, the market share of YAs is relatively low because of limited deep processing. Therefore, understanding the influence of processing conditions on the composition and function of YAs is important for production and sales, as high mineral content in food does not necessarily guarantee high absorptivity.

In this study, the effect of different types of lactic acid bacteria on the absorption rate of minerals during fermentation was analyzed using a Caco-2 cell model *in vitro* and a zebrafish model *in vivo*. Consumer attitudes toward and acceptability of YAs were also investigated. We found that the content of soluble substances and the absorption rate of minerals increased with decreasing pH during fermentation. The heterolactic lactic acid bacteria performed better than the heterolactic lactic acid bacteria in terms of the absorptivity of the minerals in YA they produced.

The minimum intake of essential minerals is related to a lack of micronutrients, whereas their maximum intake is associated with an increased risk of developing chronic diseases (30). In this study, we determined the total calcium, iron, zinc, magnesium, and phosphorus contents in soybean milk. We found the content of Ca (476.01 μg/ml), Fe (724.32 μg/ml), Zn (19.88 μg/ml), Mg (840.15 μg/ml), and P (2259.37 μg/ml) and the other soluble contents in soybean milk were increased during the fermentation, which may be due to the following four reasons. First, phytase converts calcium and magnesium phytate into calcium, magnesium ions, inositol, and inorganic phosphorus, consequently increasing the soluble content in soybean milk (31). Second, *L. plantarum* uses the carbon source in soybean milk to produce organic acids during fermentation, which reduces the pH, leading to charge neutralization or a shielding effect on the protein surface. Most negatively charged proteins are converted to positively charged (32). Thus, calcium and magnesium ions bound to the proteins were released, leading to an increase in soluble content in soybean milk. Third, the pH in YA reached 4.2–4.5 after fermentation, which is lower than the pK value of calcium and magnesium. Some calcium and magnesium organic compounds bound by chemical bonds dissociate into their ionic forms. Fourth, the lactic acid bacteria adsorb metallic elements. However, as the pH decreases, the competitive binding effect of H^+^ occurs at the position where lactic acid bacteria and calcium ions combine; thus, calcium ions are released by dissociation, and the content of water-soluble calcium ions increases. Consistent with these findings, the results of a previous study have shown that lactic acid bacteria starter cultures improved the calcium content in whole meal rye flour bread (33). Furthermore, the pre-fermentation organic phosphorus was significantly below the control levels, which rose above the control levels at the post-fermentation stage. This indicated that the production and consumption of phosphorus affect the fluctuations in water-soluble phosphorus during fermentation.

The speciation and absorptivity of the minerals determine their final bioavailability in foods (13). Here, to investigate the absorptivity, transformation, and speciation of minerals in soybean milk regulated by different types of bacterial strains, we constructed a Caco-2 cell-based intestinal barrier absorption model. The results showed that Calcium, Magnesium, Ferric, Zinc, and Phosphorus absorption rates increased during fermentation, especially at 4 h after fermentation when the pH of the protein was lower than its isoelectric point. The absorption rate reached a maximum at 8–12 h after fermentation and was stable when the microbial enzyme production activity was the highest. This is consistent with the change in the water-soluble mineral content. The increase in the calcium absorption rate may be caused by the change in quantity and the increase in soluble calcium content, on the one hand, and the change in the form of calcium, on the other hand. To better analyze the contribution of the content and mineral form to the mineral absorption rate, their speciation was found to be converted from giant molecules to small molecules along with the continuous decline of pH. This increment was time-dependent for bacterial strains cultured for 0–12 h, indicating that bacterial strains induced an increase in mineral and trace element absorptivity by converting their speciation. We speculated that this was due to the protein being broken down into peptides, which could improve the absorption of minerals through mineral–peptide complexes. Sun et al. (34) isolated a pentapeptide (DHTKE) from egg white hydrolysates and reported that the DHTKE–calcium complex improved calcium absorption by more than seven times. Sato discovered that casein phosphopeptides from cow milk resulted in more effective calcium absorption upon chelation with calcium through the phosphate groups of serine, forming soluble complexes (35). Compared to soybean milk, an unprecedented increase in absorption was observed, as stated above, which was influenced by its poor content in soybean milk. In addition, we deduced that the coordination force with minerals was weak in soybean milk.

Different metabolic types of lactic acid bacteria have different effects on the absorption rate. Homotypic lactic acid bacteria showed a higher conversion ratio during fermentation. During lactic acid fermentation, the protein was degraded through a complex proteolytic system (36). In general, the proteolytic system consists of serial chain reactions, which are produced from a serine proteinase that is proline-specific and linked to the cell wall, followed by the specific transport of dipeptides, tripeptides, and oligopeptides, and finally, by the action of a countless number of intracellular peptidases and aminopeptidases (37). In the present study, *L. plantarum* generated a higher level of calcium absorption than *E. faecalis*. This could be because *Lactobacillus* strains act with better adhesion ability, involved in the process of calcium absorption process (38). Furthermore, *Lactobacillus* strains induce the expression of calcium-absorbing host protein by promoting *MUC3* mRNA transcription and translation, improving calcium absorption (39). Additionally, these differences may have resulted from the differences in the metabolic pathways of the strains. Kleerebezem et al. (40) found that *L. plantarum* lacks genes encoding cell wall hydrolases but is abundant in peptide transport systems: oligopeptide permease transporter (pp) and dipeptide and tripeptide transporter (DtpT), which can transport extracellular peptides into cells; meanwhile, many peptidases in cells can further degrade peptides (40). A study reporting the whole genome sequence of *L. plantarum* strain 5–2 isolated from fermented soybeans identified genes encoding transport systems and intracellular peptidases with different characteristics; however, the study could not identify genes encoding cell wall proteases (41).

Changes in the structure of the protein could affect its binding ability to minerals, which changes with pH. This study used SDS-PAGE, infrared spectroscopy, and fluorescence spectroscopy to analyze protein structure changes during fermentation. Differential chromatograms indicated that the intensity of the peaks corresponding to low-molecular-mass species increased with longer fermentation times, whereas the intensity of the peaks corresponding to high-molecular-mass species diminished. A peptide with a molecular weight between 1 and 3 kDa is generally considered a small peptide, whereas that with less than 1 kDa is considered an amino acid or small-molecule substance. The peptide Glu-Gly from whey protein hydrolysates was recently identified as a strong calcium binder (42). Similarly, Asp-Glu dipeptides have been found to bind calcium more strongly than predicted by these two amino acids. In addition, it has been proved that some amino acids are potential mineral-binding ligands, such as aspartic acid and glutamic acid (34). Based on the above morphological distribution, we concluded that the YAs contained many small peptides and amino acids that could chelate minerals.

In addition, the mineral nutrients in processed foods are not directly converted into an absorbable form for human needs. For example, several ligands like phytates, oxalates, and dietary fibers may bind with minerals and reduce the bioavailability of these nutrients (43). Changes in organic matter content have dual effects on the bioavailability of minerals in soybean milk. The binding state of hydrophilic organic acids is easily transformed into an applicable state, which is affected by physical and chemical properties (pH, redox potential (Eh), organic matter content), and other factors during the fermentation process. The high content of organic matter will combine with mineral elements to form a complex and then become soluble small-molecule organic matter, thus increasing absorptivity. Heterogeneous mixtures of soluble organic molecules (proteins and polysaccharides) and various functional groups (carboxyl, phenolic, and amino) have different affinities for mineral elements. Therefore, to determine the potential reason for the increased absorption, the organic acid content was determined. Generally, the organic acid content shows a significant decrease with decreasing pH, indicating that pH plays an essential role in transforming the valence states and forms of mineral elements.

Both the quality and demand of consumers affect product sales (44). To better understand market sales, the knowledge of consumers on YAs and yogurt and acceptance of beany flavors were observed. All consumers participating in the study indicated a link to fermented food, although the frequency of consumption varied. An increased frequency of yogurt consumption increases the acceptability scores for YAs. However, the beany smell would affect purchase desire, while more details about fungi in YAs would increase purchasing power.

The absorbed calcium ions can improve bone density after entering the body. To better understand mineral absorption *in vivo*, a zebrafish animal model was used to analyze bone density and neuroactivity. The results confirmed that 100 mg/L yogurt significantly promoted the formation of new bone (*p* < 0.05), which was much better than soybean milk. Similarly, mineral and trace elements significantly improve the nervous system, enhancing swimming distance in zebrafish.

## 5. Conclusion

In this study, the effects of homotypic (*Enterococcus faecalis*) and heterolactic (*Lactiplantibacillus plantarum* CICC 21022) lactic acid bacteria fermentation on the nutritional and mineral bioavailability of SYA was investigated. The Caco-2 cell model showed the potential of fermentation to improve the acidic amino acid and organic acid contents as well as mineral absorptivity in SYA. The fermentation altered the speciation of minerals from a large molecular type to a small one. Furthermore, the chemical composition changes revealed that peptide calcium might be the main mineral form resulting from fermentation. YA substantially increased the bone mass in a zebrafish osteoporosis model. The analyses in the zebrafish model showed that YA could effectively improve the bioavailability of minerals and trace elements, further highlighting the potential of lactic acid bacterial fermentation for mineral bioavailability. Overall, this study provides evidence that lactic acid bacteria regulates the form and bioavailability of minerals and trace elements in soybean milk during fermentation. The results provide a foundation for understanding the influence of processing conditions on the composition and function of YAs and can assist in the production and sales of SYA.

## Data availability statement

The original contributions presented in the study are included in the article/Supplementary material, further inquiries can be directed to the corresponding authors.

## Author contributions

JG were responsible for conceptualizing, designing this study, validation, data curation, and writing original draft preparation. XK, KW, YuC, MD, and JX played an important role in software and formal analysis. BX and ZW participated in investigation and visualization. YoC and TY contributed to resources, writing review and editing, supervision, project administration, and funding acquisition. All authors contributed to the article and approved the submitted version.

## Funding

The work was supported by the National Science Foundation of China (31871450) and the Natural Science Foundation of Shandong (ZR2022MC217) granted its approval to conduct this research. In addition, the Center for Mitochondria and Healthy Aging, College of Life Sciences, Yantai University partly provided support.

## Conflict of interest

The authors declare that the research was conducted in the absence of any commercial or financial relationships that could be construed as a potential conflict of interest.

## Publisher’s note

All claims expressed in this article are solely those of the authors and do not necessarily represent those of their affiliated organizations, or those of the publisher, the editors and the reviewers. Any product that may be evaluated in this article, or claim that may be made by its manufacturer, is not guaranteed or endorsed by the publisher.
