# Supplementary figures and images for: Effect of fermentation using different lactic acid bacteria strains on the nutrient components and mineral bioavailability of soybean yogurt alternative
Source: Front Nutr. 2023 Jun 23;10:1198456. doi: 10.3389/fnut.2023.1198456 (PMC10327429; doi:10.3389/fnut.2023.1198456)

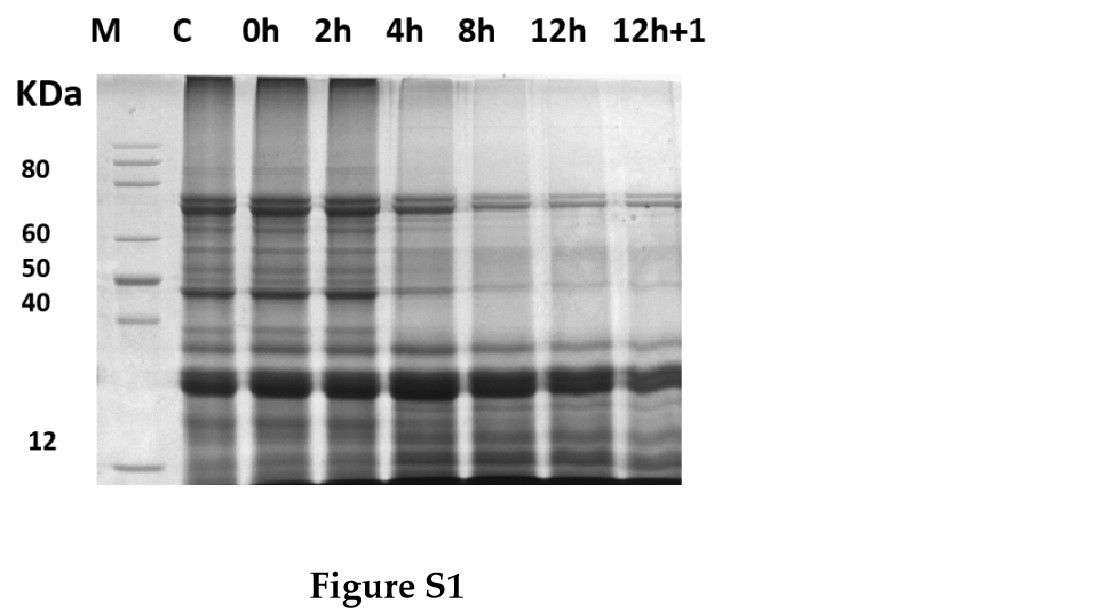

Supplement: Supplementary file 1 [file Image_1.jpeg]

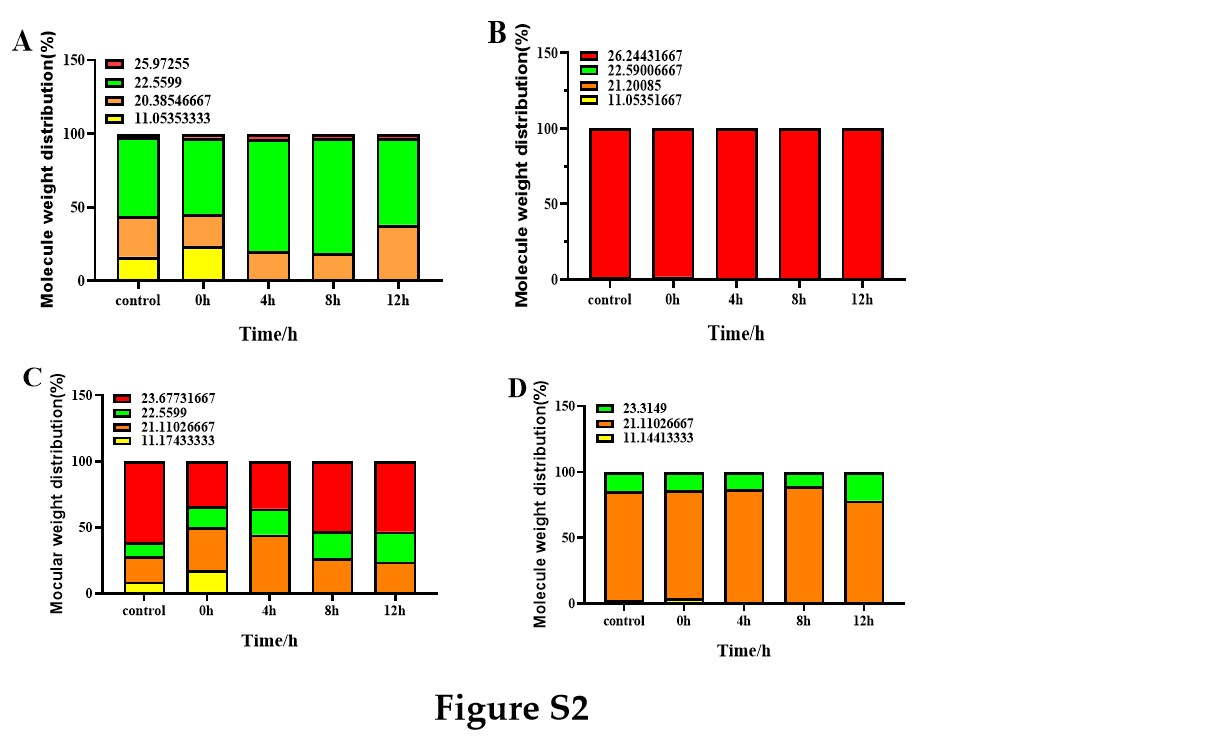

Supplement: Supplementary file 2 [file Image_2.jpeg]
